# Assessing Cognitive Behavioral Therapy for Insomnia to Improve Sleep Outcomes in Individuals With a Concussion: Protocol for a Delayed Randomized Controlled Trial

**DOI:** 10.2196/38608

**Published:** 2022-09-23

**Authors:** Rebecca Ludwig, Michael Rippee, Linda J D’Silva, Jeff Radel, Aaron M Eakman, Jill Morris, Michelle Drerup, Catherine Siengsukon

**Affiliations:** 1 Physical Therapy, Rehabilitation Science, and Athletic Training University of Kansas Medical Center Kansas City, KS United States; 2 Department of Neurology University of Kansas Medical Center Kansas City, KS United States; 3 Department of Physical Therapy and Rehabilitation Science University of Kansas Medical Center Kansas City, KS United States; 4 Department of Occupational Therapy and Therapeutic Science University of Kansas Medical Center Kansas City, KS United States; 5 Department of Occupational Therapy Colorado State University Fort Collins, CO United States; 6 Cleveland Clinic Neurological Institute, Sleep Disorders Center Cleveland, OH United States

**Keywords:** sleep, concussion, cognitive behavioral therapy, CBT, insomnia, brain, injury, RCT, randomized controlled trial, protocol, recovery, pilot study

## Abstract

**Background:**

Sleep disturbances post concussion have been associated with more frequent and severe concussion symptoms and may contribute to poorer recovery. Cognitive behavioral therapy for insomnia (CBT-I) is an effective treatment for insomnia; however, it remains unclear if this treatment method is effective in improving sleep outcomes and reducing concomitant postconcussion symptoms.

**Objective:**

The hypotheses for this study are that (1) CBT-I will improve sleep outcomes and (2) CBT-I will improve concomitant postconcussion symptoms.

**Methods:**

In total, 40 individuals who are within ≥4 weeks of postconcussion injury and have insomnia symptoms will be enrolled in this randomized controlled trial. Participants will be randomized into either a group that starts a 6-week CBT-I program immediately after baseline or a waitlist control group that starts CBT-I following a 6-week waiting period. All participants will be reassessed 6, 12, and 18 weeks after baseline. Standardized assessments measuring sleep outcomes, postconcussion symptoms, and mood will be used. Linear regression and *t* tests will be used for statistical analyses.

**Results:**

Enrollment of 40 participants was completed July 2022, data collection will be completed in November 2022, and publication of main findings is anticipated in May 2023. It is anticipated that participants experience reduced insomnia symptoms and postconcussion symptoms following CBT-I and these improvements will be retained for at least 12 weeks. Additionally, we expect to observe a positive correlation between sleep and postconcussion symptom improvement.

**Conclusions:**

Successful completion of this pilot study will allow for a better understanding of the treatment of insomnia and postconcussion symptoms in individuals following a concussion.

**Trial Registration:**

ClinicalTrials.gov NCT04885205; https://clinicaltrials.gov/ct2/show/NCT04885205

**International Registered Report Identifier (IRRID):**

DERR1-10.2196/38608

## Introduction

### Background

Between 1.7 and 3.8 million people in the United States experience a concussion each year, and the prevalence of concussions continues to increase [1-3]. A concussion injury results from a rotational or linear force to the head, neck, or face, causing injury to the brain [2,4]. Injuries from a concussion result in a number of symptoms termed *postconcussion symptoms* [5], which can be categorized into 4 different domains: somatic, cognitive functioning, mood regularity, and sleep dysregulation [5].

Sleep dysregulation is a risk factor for prolonged recovery. One recent systematic review found that poor sleep was predictive of poor long-term outcomes in the acute phase of concussion recovery [6], while another systematic review found that poor sleep in the chronic phase of recovery was associated with poor cognitive functioning (executive function and working memory) and emotional regulation [7]. Currently, it is unknown if treatment for sleep disturbances in individuals with a concussion could impact recovery.

The causes of sleep disturbances are multifactorial. Axonal damage that occurs during the concussion injury can result in dysregulation of the sleep and arousal centers in the brain [6,8-11]. Furthermore, medication use, new onset or increase in anxiety or depression, or a change in routine and the sleep schedule can contribute to sleep disturbances [6,12,13]. Sleep complaints can be present immediately following a concussion or within the first few days or weeks following the injury. The most reported sleep disturbance in individuals with a concussion, insomnia, is experienced by nearly 50% of those individuals [14]. Chronic insomnia is defined as difficulty in falling asleep or staying asleep 3 or more days a week for more than 3 months [15,16].

Chronic insomnia following a concussion can result in elevated plasma levels of neurofilament light (NfL) and tau biomarkers [17]. Both NfL and tau biomarkers have been associated with axonal damage, neuronal injury, and neurodegeneration [18]. Additionally, these biomarkers have been associated with cognitive decline and cognitive impairment [18]. Emerging research has found higher levels of plasma NfL and tau biomarkers in individuals with a history of a concussion, who also have poor sleep [19]. A possible mechanism for why these biomarkers are elevated could be disruption in the regular sleep function of metabolic waste clearance through the glymphatic system [19]. It is hypothesized that by improving sleep, metabolic waste clearance within the glymphatic system will be enhanced, which could decrease the accumulation of the NfL and tau biomarkers.

To assist in improving insomnia symptoms, cognitive behavioral therapy for insomnia (CBT-I) is the recommended first-line treatment. CBT-I consists of a multicomponent program that includes cognitive and behavioral strategies targeted to address the perpetuating factors of insomnia [20]. CBT-I is more effective than pharmaceuticals for long-term treatment of insomnia [21,22]. Systematic reviews [23,24] and meta-analyses [25,26] have found that CBT-I has a medium to large effect size in various comorbid populations and diagnoses. A recent scoping review reported that cognitive behavioral therapy (CBT) improved sleep efficiency and sleep quality and reduced insomnia symptoms in individuals with traumatic brain injury of all severities [27]. Furthermore, there was a reduction in concomitant symptoms, specifically anxiety and depression, after completion of the sleep intervention [27].

To date, only one study has evaluated the use of CBT-I in individuals who sustained a concussion [28]. The main findings from this study (n=24) indicated that CBT- I improved insomnia symptoms and sleep quality and decreased dysfunctional beliefs about sleep in adolescents but did not improve anxiety, depression, and postconcussion symptoms following CBT-I. However, a small sample size limits the interpretation of these results. Therefore, an adequately powered clinical trial is needed to evaluate if CBT-I enhances sleep outcomes in people with a concussion and impacts the recovery process.

### Objectives and Hypotheses

#### Aim 1: To Assess the Therapeutic Effect of CBT-I in Individuals With a Subacute Concussion and Symptoms of Insomnia on Sleep Outcomes

We hypothesize that CBT-I will result in a greater magnitude of change in insomnia severity and sleep quality compared to the waitlist control (WLC). Furthermore, we hypothesize that the magnitude of change in insomnia severity and sleep quality will be maintained for 12 weeks following CBT-I.

#### Aim 2: To Assess the Therapeutic Effect of CBT-I in Individuals With a Subacute Concussion and Symptoms of Insomnia on the Severity and Number of Postconcussion Symptoms, Anxiety, and Depression

We hypothesize that CBT-I will result in a greater magnitude of change in the severity and number of postconcussion symptoms, anxiety, and depression compared to the WLC.

#### Aim 3: To Evaluate the Relationship Between Improvement in Sleep Outcomes and Postconcussion Symptoms

We hypothesize that improvement in insomnia severity will be positively associated with reductions in the severity and number of postconcussion symptoms.

#### Exploratory Aim: To Evaluate the Therapeutic Effect of CBT-I in Individuals With a Subacute Concussion and Symptoms of Insomnia on Levels of NfL and p-tau Biomarkers.

We hypothesize that there will be a significant reduction in plasma NfL and p-tau levels from baseline to post CBT-I.

## Methods

### Study Overview

**Figure 1:**
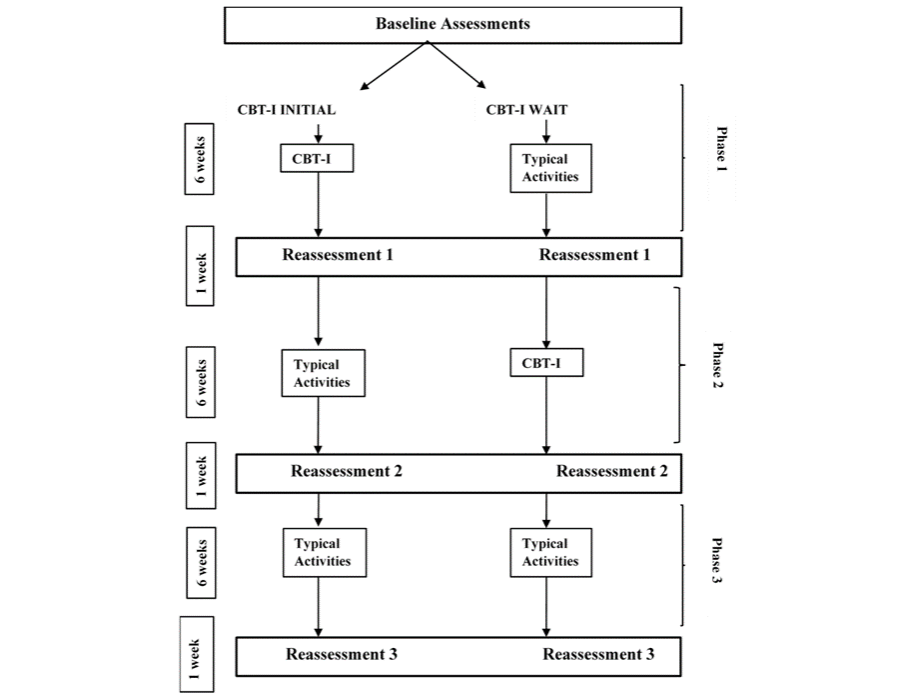
Study flowchart. CBT-I: cognitive behavioral therapy for insomnia.

The proposed study is a delayed-start randomized controlled trial of 6 weeks of CBT-I among individuals with a concussion, aged 18-64 years old (n=40; Figure 1). Individuals who meet the inclusion criteria (Textbox 1) will be randomized into 2 groups. The first is a CBT-I initial group (CBT-I initial; n=20), which will start the CBT-I intervention directly after completing the baseline assessment. The second group is the WLC group (n=20), which will start the CBT-I intervention 6 weeks after the baseline assessment. The final 6 weeks for all participants, regardless of group assignment, will include participation in typical activities such as maintaining employment status, family roles, etc. 

**Inclusion and exclusion criteria.**

**Inclusion criteria:**

- Aged 18 to 64 years
- Within ≥4 weeks of concussion injury
- Self-report difficulty falling asleep, maintaining sleep, or waking up too early at least 3 nights per week since injury
- Score of ≥10 on the Insomnia Severity Index to indicate clinical insomnia [29]
- Score of ≥17 on the Mini-Mental State Examination-Telephone questionnaire [30]

**Exclusion criteria:**

- Known untreated sleep disorder (such as sleep apnea, restless leg syndrome, circadian rhythm disorder, hypersomnia, or parasomnias)
- Increased risk obstructive sleep apnea (score≥3 on the snoring, tiredness, observed apnea, high BP, BMI, age, neck circumference, and male gender questionnaire) [31,32]
- Increased risk of restless leg syndrome on the Restless Leg Syndrome Diagnosis Index [33]
- Increased risk of circadian rhythm sleep-wake disorder [34]
- Increased risk of parasomnia [34]
- Active abuse or history (up to 2 years) of alcohol or drug dependence as defined by the Diagnostic and Statistical Manual of Mental Disorders, Fifth Edition criteria [35]
- Severe mental illness such as schizophrenia or bipolar disorder
- Score of >29 on the Beck Depression Inventory or indication of suicidality (response of “2” or “3” to item 9) [36]
- History of diagnosed nervous system disorders other than concussion (such as multiple sclerosis, Parkinson disease, or stroke)
- Currently works in a night shift

### Ethical Considerations

This study was approved by the institutional review board at the University of Kansas Medical Center (STUDY00146439) and was conducted in accordance with the ethical standards of the Helsinki Declaration.

### Recruitment

Recruitment will primarily be carried out through physician referral at the concussion management clinic at the University of Kansas Medical Center (KUMC). Potential participants will also be contacted through the KUMC Heron Data Repository/Pioneers participant registry [37]—a registry of patients from the University of Kansas Health System who have given consent to be contacted for potential research. If the number of participants cannot be reached through these primary methods, participants will be sought from other concussion clinics in the Kansas City area, in community support groups, and through social media.

### Screening Procedures

Individuals will undergo a 2-step screening process. The first portion will consist of a standard telephone screening, which includes a review of the inclusion and exclusion criteria (Textbox 1); Insomnia Severity Index (ISI) [29] to assess insomnia symptom severity; snoring, tiredness, observed apnea, high BP, BMI, age, neck circumference, and male gender questionnaire to assess risk of sleep apnea [31,32]; the Restless Leg Syndrome-Diagnostic Index to assess for risk of restless leg syndrome [33]; and the Clinical Interview for Sleep Disorders-Revised [34] questionnaire to assess for circadian rhythm disorders, nightmare disorder, night terror disorder, and REM sleep behavior disorders. The Mini-Mental State Examination Telephone [30,38] will be administered for a cognition screen for informed consent purposes. The second step to the screening process will be to administer the Beck Depression Inventory (BDI) [36] by emailing the potential participant a link to complete the BDI using the Research Electronic Data Capture (REDCap) database [39,40]. The principal investigator, RL, will review the completed BDI to ensure eligibility. If participants are eligible, a link to complete the consent process via REDCap will be emailed to each prospective participant. After the consent process is completed, the participant will be emailed a link to complete the baseline assessments.

### Assessments

#### Overview

The baseline assessments will be sent via a REDCap survey within 2 days of the main consent completion. The baseline evaluation will consist of demographic information collection and a completion of questionnaires to assess sleep outcomes, postconcussion symptoms, and mood.

#### Demographics

The following demographic data will be collected via self-report during baseline assessment: sex, education level, ethnicity, race, marital status, number of physician-diagnosed concussions, mechanism of concussion injury, list of current medications and dosage of those medications, list of health care services currently receiving, and a question asking if insomnia started before or after the concussion injury.

#### Sleep Questionnaires

The ISI [29] is a valid and reliable measure of insomnia severity that consists of 7 questions, each rated on a scale of 0-4. The range of scores on the ISI is 0-28; a score of ≥10 suggests clinical insomnia [41]. The ISI completed at screening will be considered the baseline ISI score.

The Pittsburgh Sleep Quality Index (PSQI) will be used to assess quality of sleep [42]. Scores range from 0-21, with a higher score indicating lower quality of sleep. A global score of > 5 indicates poor sleep quality.

#### Postconcussion Symptom Severity Questionnaires

Postconcussion symptom severity and the number of symptoms will be assessed using the Post-Concussion Symptom Scale (PCSS) [43]. The severity of 22 concussion-related symptoms is assessed on a Likert scale ranging 0-6 with 0=“no symptom” and 6=“extreme symptom” [44]. A score of 132 is the maximum, indicating that all symptoms are severe [44]. The number of postconcussion symptoms will be assessed by counting the number of symptoms that the participant identified as having a severity of 1 or higher on the PCSS. A score of a 1 indicates that the symptom is mild but still noticeable.

#### Mood Questionnaires

The BDI [36] assessment will be used to assess the level of depression. The tool consists of 21 items that are scored on a Likert scale of 0-3 with 0=“no change in symptom” and 3=“severe change in symptom.” Scores range from minimal depression (0-13), mild depression (14-19), moderate depression (20-28), and severe depression (>29).

The BDI completed during screening will yield the baseline BDI score. The Beck Anxiety Inventory (BAI) [45] assessment will be used to assess the level of anxiety. The tool consists of 21 items that are scored on a Likert scale of 1-3 with 0=“not at all” and 3=“severely.” Scores range from minimal anxiety (0-7), mild anxiety (8-15), moderate anxiety (16-25), and severe anxiety (>30).

#### Blood Plasma Biomarkers

Blood plasma biomarkers will be assessed at baseline for all individuals enrolled and within 1 week of completing the CBT-I intervention. A trained phlebotomist will draw blood (~10 mL) into a vacutainer tube containing EDTA as an anticoagulant. The EDTA tube will then be inverted several times to mix prior to centrifugation at 1500 *g* for 10 minutes at 4 °C. After processing, plasma will be transferred into aliquots and stored at –80 °C until analysis. NfL and p-tau analysis will use Single Molecule Array analytics (Quanterix).

#### Reassessments

Reassessments will occur 6, 12, and 18 weeks following baseline. Participants will update their current medications and health care services. Additionally, sleep questionnaires, concussion symptoms, and mood questionnaires completed during baseline will be completed at reassessments.

### Randomization

Following the baseline assessment, participants will be randomized to either the CBT-I initial group (n=20) or the CBT-I WLC group (n=20; Figure 1). A randomization list was developed using SPSS [46]. Participants are not blinded to their group assignment. One researcher (RL) is providing the treatment for this study and is not blinded to group allocation. Questionnaires are sent to participants via REDCap, and participants will be able to answer the questionnaires on their own time and in their own environment. The same researcher (RL) is also performing data analyses for this study.

### The CBT-I Intervention

#### Overview

The CBT-I program is a 6-week, in-person, one-on-one program conducted by RL, a trained CBT-I provider, and with consultation from a Diplomate in Behavioral Sleep Medicine. The CBT-I will be delivered remotely over a Health Insurance Portability and Accountability Act–compliant teleconference service (Zoom) or via telephone. A standardized CBT-I program will be used [47]. Participants will be given a sleep log to maintain throughout the course of the 6-week intervention. The sleep log will be used to tailor the intervention and to monitor adherence to the intervention for each participant. Each weekly session will start with a discussion on the previous week of sleep, review of the sleep diary, and the prior week’s goals. The general session outlines are as follows with each session lasting approximately 45-60 minutes.

#### Session 1

This session involves gaining a qualitative sleep history, establishing a treatment plan, establishing a sleep schedule and stimulus control, collaborating on strategies for how to stay awake to the prescribed bedtime and what to do if the participant wakes up in the middle of the night, and discussing healthy sleep practices as they relate to a concussion.

#### Session 2

This session involves reviewing the concepts of circadian rhythm and stimulus control, asking about adherence with possible adjustment to sleep compression bedtime and wake times, and introducing the relaxation techniques of diaphragmatic breathing and deep breathing and their application in daily living.

#### Session 3

This session involves reinforcing concepts of the circadian rhythm and stimulus control, continuing to monitor the prescribed sleep compression bedtime and wake time, adjusting bedtimes and wake times accordingly, and introducing the relaxation technique of mindfulness and its application in daily living.

#### Session 4

This session involves continuing to reinforce the ideas of circadian rhythm and stimulus control while monitoring the prescribed sleep compression bedtime and wake time, adjusting bedtimes and wake times accordingly, and introducing the relaxation technique of progressive muscle relaxation and its application in daily living.

#### Session 5

This session involves discussing negative sleep beliefs or reinforcing a relaxation technique from previous sessions.

#### Session 6

This session involves discussing the sleep log and determining if adjustments are needed to the sleep schedule, reviewing the prior week’s goals, assessing global treatment gains, and discussing relapse prevention.

#### WLC Activities

During the wait period portion, the WLC group will be encouraged to continue participation in their typical activities including employment, appointments, and personal schedule. After the 6-week wait period, the WLC group will receive the full CBT-I intervention.

### Statistical Analysis

#### Sample Size

For this study’s power analysis, the effect size is set at 0.8, which is based on a prior study that had large effect sizes on insomnia (0.8) [28]. The SD is set at 1 and the α is set at .05. Therefore, the required sample size is 32 participants, which is anticipated on the basis of clinical experience that 10%-25% of participants will drop out of the study so an additional 8 participants will be enrolled; thus, 40 participants will be enrolled for a total of 20 participants in each group. Intention-to-treat analyses will be used; hence, in the event of a participant dropping out after randomization, the data from their last assessment will be carried forward to the reassessment [48].

#### Statistical Approach

##### Aim 1: To Assess the Therapeutic Effect of CBT-I in Individuals With a Subacute Concussion on Insomnia Symptoms and Sleep Quality

An independent samples *t* test will be used to determine if there is a significant difference in percent change in the ISI (primary outcome) and PSQI between the CBT-I and the WLC conditions. To assess maintenance for each group, paired *t* tests will be used to determine if there is a significant difference at the following time points: from the reassessment following CBT-I (reassessment 1 for the CBT-I group; reassessment 2 for the WLC group) to the immediate reassessment following the typical activities period (reassessment 2 for the CBT-I initial group; reassessment 3 for the WLC group), and from reassessment following CBT-I to delayed reassessment (reassessment 1 to reassessment 3 for CBT-I group). The percentage of participants who meet the minimal clinically important difference (MCID) will also be reported (7 points for the ISI [36] and 3 points for the PSQI [39]). In the event that the data do not meet the assumptions for parametric *t* tests, nonparametric tests (Mann-Whitney *U* test for independent samples, and Wilcoxon signed rank test for paired samples) will be used.

##### Aim 2: To Assess the Therapeutic Effect of CBT-I in Individuals With a Subacute Concussion on the Severity and Number of Postconcussion Symptoms, Anxiety, and Depression

An independent samples *t* test will be used to determine if there is a significant difference in percent change for the PCSS, BAI, and BDI between the CBT-I condition and the WLC group. To assess maintenance, paired samples *t* tests will be used to determine if there is a significant difference from the following time points: from the reassessment following CBT-I (reassessment 1 for the CBT-I group; reassessment 2 for the WLC group) to the immediate reassessment following the typical activities period (reassessment 2 for the CBT-I Initial group; reassessment 3 for the WLC group), or from reassessment following CBT-I to delayed reassessment (reassessment 1 to reassessment 3 for the CBT-I group). The percentage of participants who meet the MCID criteria for each outcome will be reported (12 points for the PCSS, 4 points for the total number of symptoms on the PCSS, 4 points on the BAI, and 5 Points on the BDI [40,45,46]). In the event that the data do not meet the assumptions for parametric *t* tests, nonparametric tests (Mann-Whitney *U* test for independent samples and Wilcoxon signed rank test for paired samples) will be used.

##### Aim 3: To Evaluate the Relationship Between Improvement in Sleep Outcomes and Postconcussion Symptoms

Two simple linear regressions will be run to evaluate the relationship between improvement in sleep outcomes and postconcussion symptoms. The first simple linear regression will determine if change in ISI from preintervention to postintervention stages (predictor variable) will predict changes in postconcussion severity (dependent variable). The second simple linear regression will determine if change in the ISI from preintervention to postintervention stages (predictor variable) will predict changes in the number of postconcussion symptoms (dependent variable). If there are significant differences at baseline in age, gender, medication use, number of concussions, and receiving other services between the two groups, the variables will be entered into the model as a covariate, and the 2 models will be rerun.

##### Exploratory Aim: To Evaluate the Therapeutic Effect of CBT-I in Individuals With a Subacute Concussion and Symptoms of Insomnia on Levels of NfL and p-Tau Biomarkers

Paired samples *t* tests will be used to determine if there is a significant change in NfL and p-tau plasma levels before and after the CBT-I intervention. In the event that the data do not meet the assumptions for parametric *t* tests, nonparametric tests (Mann-Whitney *U* test for independent samples and Wilcoxon signed rank test for paired samples) will be used.

## Results

Enrollment of 40 participants was completed in July 2022, data collection is anticipated to be completed in November 2022, and publication of main findings is anticipated in May 2023. After final data analysis and writing of the results, the manuscripts will be submitted to appropriate journals for dissemination. It is expected that participants in this study will experience a reduction in insomnia symptoms and an increase in sleep quality after CBT-I, and these improvements in insomnia symptoms and sleep quality will be retained following the intervention. Additionally, it is expected that the therapeutic effect of CBT-I will result in an improvement in postconcussion symptom severity and the number of symptoms. Improvement in insomnia severity will predict improvement in the severity and number of postconcussion symptoms. It is also anticipated that there will be a reduction in plasma NfL and p-tau levels following the CBT-I intervention.

## Discussion

### Expected Findings

It is anticipated that individuals who complete CBT-I will have improved sleep outcomes as well as reduced postconcussion symptoms. Additionally, it is anticipated CBT-I will reduce NfL and p-tau levels. This study may modify the traditional approach to postconcussion care by asserting that insomnia can be resolved by CBT-I and that improvement in insomnia symptoms is associated with improvement in postconcussion symptoms. The use of CBT-I in individuals with a concussion is relatively novel as there is currently only one published pilot randomized controlled trial [28]. Additionally, the biomarker substudy will provide valuable insights into the mechanisms of how sleep assists in neuronal recovery. This study provides both a mechanistic and clinical perspective for the treatment of individuals with postconcussion symptoms.

The number and severity of symptoms following a concussion vary among individuals [49,50,51,52]. In the majority of concussion cases, 80%-90% of individuals experience symptom resolution between 1 and 3 weeks post injury [1,4,5,53]. However, for the remaining 10%-15% of concussion cases, symptoms persist for months to years post injury [54-57]. Combined, approximately 50% of individuals recovering from a concussion have insomnia symptoms [14], and 10%-15% of people have prolonged recovery. Therefore, there is a high percentage of individuals who would benefit from standard use of CBT-I to resolve their insomnia symptoms and mitigate their postconcussion symptoms.

This study also offers a potential insight into the mechanism of how improvement in sleep assists in neuronal recovery and the possible prevention of neurodegeneration. One marker for recovery could be the presence of NfL in p-tau as these markers are associated with neuronal injury and neurodegeneration. Further exploration of the presence of these biomarkers may provide valuable information on how significant symptoms of insomnia affect recovery and how those biomarker levels respond with improvement in sleep following CBT-I intervention.

### Anticipated Limitations

Potential limitations related to the treatment response are as follows. First, it is unknown how each participant will respond to the CBT-I treatment with their current symptom burden. Enrolling participants who are within at least 4 weeks of their concussion was to obtain individuals who have relatively stable symptoms at the time of enrollment. Second, medication use and dosage may affect the treatment response to the CBT-I intervention. Therefore, information on medication will be collected at baseline and at reassessments to be included in statistical analysis if indicated. Third, participants will potentially be receiving care from other health care providers, which could impact the interpretation of the results. Therefore, information on type of services being received will be collected at baseline and at reassessments to be included in statistical analysis if indicated.

Another potential limitation to this study is attrition. The attrition rate was 20% in one study evaluating CBT-I in adolescents with a concussion [28]. A 20% attrition rate was also observed in a cognitive behavioral intervention study on adults who have had a concussion [58]. To account for attrition, we will overenroll by 20% in this study. To help with retention, study procedures and time commitment will be clearly articulated to potential participants and reinforced during the course of the study. Furthermore, individuals in the WLC will be contacted by telephone in the middle of their 6-week waiting period to remind them of their upcoming CBT-I sessions and to maintain connection and interest.

### Conclusions

Insomnia is the most common sleep disturbance in individuals recovering from a concussion. This study is the first randomized control trial to evaluate if CBT-I improves sleep outcomes and postconcussion symptoms. Potential results from this study could indicate that improvement in sleep may improve postconcussion symptoms—this could be insightful research to improve both clinical care and progress clinical research in the concussion community. Future studies need to continue to evaluate (1) the mechanistic response to improvement in sleep and (2) the relationship between improved sleep and improved recovery from postconcussion symptoms.

## Abbreviations

BAI: Beck Anxiety Inventory
BDI: Beck Depression Inventory
CBT: cognitive behavioral therapy
CBT-I: cognitive behavioral therapy for insomnia
ISI: Insomnia Severity Index
KUMC: University of Kansas Medical Center
NfL: neurofilament light
PCSS: Post-Concussion Symptom Scale
PSQI: Pittsburgh Sleep Quality Index
REDCap: Research Electronic Data Capture
WLC: waitlist control

## Data Sharing

The data sets generated and analyzed in this study may be available from the corresponding author upon reasonable request.

## References

[1] Mahooti N (2018). Sports-related concussion: acute management and chronic postconcussive issues. Child Adolesc Psychiatr Clin N Am.

[2] Duclos C, Dumont M, Wiseman-Hakes C, Arbour C, Mongrain V, Gaudreault P, Khoury S, Lavigne G, Desautels A, Gosselin N (2014). Sleep and wake disturbances following traumatic brain injury. Pathol Biol (Paris).

[3] Bramley H, Hong J, Zacko C, Royer C, Silvis M (2016). Mild traumatic brain injury and post-concussion syndrome: treatment and related sequela for persistent symptomatic disease. Sports Med Arthrosc Rev.

[4] Chancellor SE, Franz ES, Minaeva OV, Goldstein LE (2019). Pathophysiology of concussion. Semin Pediatr Neurol.

[5] McCrea MA, Nelson LD, Guskiewicz K (2017). Diagnosis and management of acute concussion. Phys Med Rehabil Clin N Am.

[6] Ludwig R, D'Silva L, Vaduvathiriyan P, Rippee MA, Siengsukon C (2020). Sleep disturbances in the acute stage of concussion are associated with poorer long-term recovery: a systematic review. PM R.

[7] Ludwig R, Nelson E, Vaduvathiriyan P, Rippee MA, Siengsukon C (2021). Sleep quality in the chronic stage of concussion is associated with poorer recovery: a systematic review. J Concussion.

[8] Wickwire EM, Williams SG, Roth T, Capaldi VF, Jaffe M, Moline M, Motamedi GK, Morgan GW, Mysliwiec V, Germain A, Pazdan RM, Ferziger R, Balkin TJ, MacDonald ME, Macek TA, Yochelson MR, Scharf SM, Lettieri CJ (2016). Sleep, sleep disorders, and mild traumatic brain injury. What we know and what we need to know: findings from a national working group. Neurotherapeutics.

[9] Parcell DL, Ponsford JL, Redman JR, Rajaratnam SM (2008). Poor sleep quality and changes in objectively recorded sleep after traumatic brain injury: a preliminary study. Arch Phys Med Rehabil.

[10] Singh K, Morse AM, Tkachenko N, Kothare SV (2016). Sleep disorders associated with traumatic brain injury-a review. Pediatr Neurol.

[11] Mollayeva T, Mollayeva S, Colantonio A (2016). The risk of sleep disorder among persons with mild traumatic brain injury. Curr Neurol Neurosci Rep.

[12] Zumstein M, Moser M, Mottini M, Ott SR, Sadowski-Cron C, Radanov BP, Zimmermann H, Exadaktylos A (2011). Long-term outcome in patients with mild traumatic brain injury: a prospective observational study. J Trauma.

[13] Ayalon L, Borodkin K, Dishon L, Kanety H, Dagan Y (2007). Circadian rhythm sleep disorders following mild traumatic brain injury. Neurology.

[14] Ouellet M, Beaulieu-Bonneau S, Morin CM (2015). Sleep-wake disturbances after traumatic brain injury. Lancet Neurol.

[15] Morin CM, Bootzin RR, Buysse DJ, Edinger JD, Espie CA, Lichstein KL (2006). Psychological and behavioral treatment of insomnia:update of the recent evidence (1998-2004). Sleep.

[16] Sateia MJ (2014). International classification of sleep disorders-third edition: highlights and modifications. Chest.

[17] Zhang P, Tan C, Chen G, Ge Y, Xu J, Xia L, Wang F, Li X, Kong X (2018). Patients with chronic insomnia disorder have increased serum levels of neurofilaments, neuron-specific enolase and S100B: does organic brain damage exist?. Sleep Med.

[18] Clark C, Lewczuk P, Kornhuber J, Richiardi J, Maréchal B, Karikari TK, Blennow K, Zetterberg H, Popp J (2021). Plasma neurofilament light and phosphorylated tau 181 as biomarkers of Alzheimer's disease pathology and clinical disease progression. Alzheimers Res Ther.

[19] Werner J, Shahim P, Pucci JU, Lai C, Raiciulescu S, Gill JM, Nakase-Richardson R, Diaz-Arrastia R, Kenney K (2021). Poor sleep correlates with biomarkers of neurodegeneration in mild traumatic brain injury patients: a CENC study. Sleep.

[20] Edinger JD, Means MK (2005). Cognitive-behavioral therapy for primary insomnia. Clin Psychol Rev.

[21] Thomas A, Greenwald BD (2018). Nonpharmacological management of sleep disturbances after traumatic brain injury. NRE.

[22] Wang M, Wang S, Tsai P (2005). Cognitive behavioural therapy for primary insomnia: a systematic review. J Adv Nurs.

[23] Wu JQ, Appleman ER, Salazar RD, Ong JC (2015). Cognitive behavioral therapy for insomnia comorbid with psychiatric and medical conditions: a meta-analysis. JAMA Intern Med.

[24] Mitchell MD, Gehrman P, Perlis M, Umscheid CA (2012). Comparative effectiveness of cognitive behavioral therapy for insomnia: a systematic review. BMC Fam Pract.

[25] Trauer JM, Qian MY, Doyle JS, Rajaratnam SM, Cunnington D (2015). Cognitive behavioral therapy for chronic insomnia. Ann Intern Med.

[26] Morin CM, Culbert JP, Schwartz SM (1994). Nonpharmacological interventions for insomnia: a meta-analysis of treatment efficacy. Am J Psychiatry.

[27] Ludwig R, Vaduvathiriyan P, Siengsukon C (2020). Does cognitive-behavioural therapy improve sleep outcomes in individuals with traumatic brain injury: a scoping review. Brain Inj.

[28] Tomfohr-Madsen L, Madsen JW, Bonneville D, Virani S, Plourde V, Barlow KM, Yeates KO, Brooks BL (2020). A pilot randomized controlled trial of cognitive-behavioral therapy for insomnia in adolescents with persistent postconcussion symptoms. J Head Trauma Rehabil.

[29] Bastien C, Vallières A, Morin CM (2001). Validation of the Insomnia Severity Index as an outcome measure for insomnia research. Sleep Med.

[30] Roccaforte WH, Burke WJ, Bayer BL, Wengel SP (1992). Validation of a telephone version of the mini-mental state examination. J Am Geriatr Soc.

[31] Chung F, Yegneswaran B, Liao P, Chung SA, Vairavanathan S, Islam S, Khajehdehi A, Shapiro CM (2008). STOP questionnaire: a tool to screen patients for obstructive sleep apnea. Surv Anesthesiol.

[32] Chung F, Abdullah HR, Liao P (2016). STOP-Bang questionnaire: a practical approach to screen for obstructive sleep apnea. Chest.

[33] Garcia-Borreguero D, Stillman P, Benes H, Buschmann H, Chaudhuri KR, Gonzalez Rodríguez VM, Högl B, Kohnen R, Monti GC, Stiasny-Kolster K, Trenkwalder C, Williams A, Zucconi M (2011). Algorithms for the diagnosis and treatment of restless legs syndrome in primary care. BMC Neurol.

[34] Structured Clinical Interview for Sleep Disorders (SCISD) and Revised (SCISD-R). Insomnia and Sleep Health Research Laboratory. The University of Arizona.

[35] (2013). American Psychiatric Association: Diagnostic and statistical manual of mental disorders, 5 ed.

[36] Beck AT (1961). An inventory for measuring depression. Arch Gen Psychiatry.

[37] Waitman LR, Warren JJ, Manos EL, Connolly DW (2011). Expressing observations from electronic medical record flowsheets in an i2b2 based clinical data repository to support research and quality improvement. AMIA Annu Symp Proc.

[38] Newkirk LA, Kim JM, Thompson JM, Tinklenberg JR, Yesavage JA, Taylor JL (2004). Validation of a 26-point telephone version of the mini-mental state examination. J Geriatr Psychiatry Neurol.

[39] Harris PA, Taylor R, Minor BL, Elliott V, Fernandez M, O'Neal L, McLeod L, Delacqua G, Delacqua F, Kirby J, Duda SN, REDCap Consortium (2019). The REDCap consortium: building an international community of software platform partners. J Biomed Inform.

[40] Harris PA, Taylor R, Thielke R, Payne J, Gonzalez N, Conde JG (2009). Research electronic data capture (REDCap)--a metadata-driven methodology and workflow process for providing translational research informatics support. J Biomed Inform.

[41] Morin CM, Belleville G, Bélanger L, Ivers H (2011). The Insomnia Severity Index: psychometric indicators to detect insomnia cases and evaluate treatment response. Sleep.

[42] Buysse DJ, Reynolds CF, Monk TH, Berman SR, Kupfer DJ (1989). The Pittsburgh sleep quality index: a new instrument for psychiatric practice and research. Psychiatry Res.

[43] Merritt VC, Bradson ML, Meyer JE, Arnett PA (2018). Evaluating the test-retest reliability of symptom indices associated with the ImPACT post-concussion symptom scale (PCSS). J Clin Exp Neuropsychol.

[44] Lau BC, Collins MW, Lovell MR (2011). Sensitivity and specificity of subacute computerized neurocognitive testing and symptom evaluation in predicting outcomes after sports-related concussion. Am J Sports Med.

[45] Beck AT, Epstein N, Brown G, Steer RA (1988). An inventory for measuring clinical anxiety: psychometric properties. J Consult Clin Psychol.

[46] IBM SPSS software. IBM.

[47] Perlis ML, Jungquist C, Smith MT, Posner D (2008). Cognitive behavioral treatment of insomnia: a session-by-session guide.

[48] Gupta S (2011). Intention-to-treat concept: a review. Perspect Clin Res.

[49] Giza CC, Hovda DA (2014). The new neurometabolic cascade of concussion. Neurosurgery.

[50] Barkhoudarian G, Hovda DA, Giza CC (2011). The molecular pathophysiology of concussive brain injury. Clin Sports Med.

[51] Frati A, Cerretani D, Fiaschi A, Frati P, Gatto V, La Russa R, Pesce A, Pinchi E, Santurro A, Fraschetti F, Fineschi V (2017). Diffuse axonal injury and oxidative stress: a comprehensive review. Int J Mol Sci.

[52] Ma J, Zhang K, Wang Z, Chen G (2016). Progress of research on diffuse axonal injury after traumatic brain injury. Neural Plast.

[53] Tator CH (2013). Concussions and their consequences: current diagnosis, management and prevention. CMAJ.

[54] Daneshvar DH, Riley DO, Nowinski CJ, McKee AC, Stern RA, Cantu RC (2011). Long-term consequences: effects on normal development profile after concussion. Phys Med Rehabil Clin N Am.

[55] Iverson GL (2019). Network analysis and precision rehabilitation for the post-concussion syndrome. Front Neurol.

[56] McMahon PJ, Hricik A, Yue JK, Puccio AM, Inoue T, Lingsma HF, Beers SR, Gordon WA, Valadka AB, Manley GT, Okonkwo DO, TRACK-TBI Investigators (2014). Symptomatology and functional outcome in mild traumatic brain injury: results from the prospective TRACK-TBI study. J Neurotrauma.

[57] Hiploylee C, Dufort PA, Davis HS, Wennberg RA, Tartaglia MC, Mikulis D, Hazrati L, Tator CH (2017). Longitudinal study of postconcussion syndrome: not everyone recovers. J Neurotrauma.

[58] Theadom A, Barker-Collo S, Jones K, Dudley M, Vincent N, Feigin V (2018). A pilot randomized controlled trial of on-line interventions to improve sleep quality in adults after mild or moderate traumatic brain injury. Clin Rehabil.

